# Psychometric Properties of the Catalan and Spanish Versions of the Anxiety, Depression and Mood Scale (ADAMS) in Adults With Intellectual Disabilities

**DOI:** 10.1111/jar.70323

**Published:** 2026-10-22

**Authors:** Albert Oliveras‐Fàbregas, Salvador Simó‐Algado, Raimon Milà‐Villaroel, Josep Oliveras‐Cuquet, Iván Sánchez‐Martínez, Maria Giné‐Garriga

**Affiliations:** ^1^ School of Psychology, Education, and Sport Sciences Blanquerna Universitat Ramon Llull Barcelona Spain; ^2^ Escuelas Universitarias Gimbernat, adscritas a la, Universitat Autònoma de Barcelona Barcelona Spain; ^3^ Grup de Recerca en Innovació en Salut Mental i Benestar Emocional (ISaMBeS), Institut de Recerca i Innovació en Ciències de la Vida i de la Salut a la Catalunya Central (IRIS‐CC) Vic Spain; ^4^ School of Health Sciences Blanquerna Universitat Ramon Llull Barcelona Spain; ^5^ Institut Universitari per a la Recerca a I'Atenció Primària de Salut Jordi Gol i Gurina (IDIAPJGol) Barcelona Spain; ^6^ Research Group on Methodology, Methods, Models and Outcome of Health and Social Sciences (M_3_O), Faculty of Health Science and Welfare, University of Vic‐Central University of Catalonia (UVIC‐UCC) Vic Spain

**Keywords:** ADAMS, cross‐cultural validation, intellectual disability, mental health, psychometrics

## Abstract

**Background:**

Adults with intellectual disabilities (ID) face an increased risk of emotional and behavioural problems but validated mental health tools are limited in Catalan and Spanish. We evaluated the Catalan and Spanish ADAMS versions in adults with ID.

**Methods:**

Proxy informants completed the ADAMS for 240 adults with mild‐to‐severe ID from community and residential services. The sample was 57.9% female, with a mean age of 36.5 years (SD = 11.9). Assessments occurred at baseline and 7 days later.

**Results:**

Internal consistency was acceptable (*α* = 0.72–0.73). The five‐factor model showed moderate fit (CFI = 0.82–0.83) and acceptable fit (RMSEA = 0.047–0.048). Test–retest reliability was excellent (CCC = 0.87–0.96), with low measurement error and no systematic bias.

**Conclusions:**

Both versions showed good reliability and temporal stability, with partial support for structural validity, supporting proxy‐based screening and monitoring in adults with ID.

## Lay Summary

- We evaluated the Catalan and Spanish versions of the ADAMS in 240 adults with intellectual disabilities.
- Both versions showed good reliability and good stability over time, although evidence for the factor structure was mixed.
- The findings support their use to screen and monitor emotional and behavioural problems in adults with intellectual disabilities.

## 1 Introduction

Adults with intellectual disabilities (ID) experience higher rates of emotional and behavioural problems and greater psychiatric comorbidity than the general population, highlighting the need for mental health assessment tools that are appropriately designed and validated for this group (Hsieh et al. 2020; Hughes‐McCormack et al. 2017).

Mood and anxiety disorders are highly prevalent among adults with ID, yet they often remain underdiagnosed or misdiagnosed because of communication difficulties, diagnostic overshadowing, and limited clinician training in ID mental health (Deb et al. 2022; Edwards et al. 2022; Havercamp et al. 2022). In practice, these challenges often require reliance on proxy informants, increasing the risk of attributing psychiatric symptoms to ID rather than recognising them as distinct and potentially treatable conditions (Santoro et al. 2022). This makes psychometrically robust instruments that are sensitive to mood and anxiety symptoms and suitable for proxy use in routine services particularly important (Maguire et al. 2023; Patel et al. 2023).

Although several instruments are available for assessing mental health in adults with ID, many still show incomplete or weak evidence for key psychometric properties such as construct validity, reliability, and measurement error (Patel et al. 2023). This gap is particularly evident in Spanish‐speaking contexts, where relatively few mental health instruments for adults with ID demonstrate strong psychometric quality (González‐Sanguino et al. 2025). Valid, culturally adapted instruments are essential for early detection of mental health problems and for supporting equitable access to services (Barrington et al. 2025; Karlsen et al. 2025).

The anxiety, depression, and mood scale (ADAMS) was developed to address the lack of comprehensive tools for assessing anxiety and depression in adults with ID (Esbensen et al. 2003). It is a 28‐item proxy‐rated questionnaire comprising five subscales: manic/hyperactive behaviour, depressed mood, social avoidance, general anxiety, and obsessive‐compulsive behaviour, which together capture observable behavioural and emotional signs of affective disturbance. Previous studies have reported acceptable reliability and validity in community and residential ID service settings in Europe and North America (Hamers et al. 2019; Hermans et al. 2012).

Beyond its psychometric performance, the ADAMS has clear clinical relevance as a practical proxy‐based instrument for identifying emotional and behavioural difficulties in adults with ID when self‐report is limited or unavailable. This is particularly valuable in residential, occupational, and community services, where caregivers and staff can observe behaviour over time and support ongoing monitoring (Esbensen et al. 2003; Witt et al. 2025). The availability of validated Catalan and Spanish versions further expands its accessibility and may facilitate culturally and linguistically appropriate assessment in adults with ID (González‐Sanguino et al. 2025; Reyes‐Martin et al. 2025).

To date, no studies have examined the psychometric properties of the ADAMS in Catalan or Spanish for adults with ID (Carbó‐Carreté et al. 2015). This limits systematic screening for anxiety and depression, as well as cross‐linguistic comparability in research and practice (McKown et al. 2020). Following international recommendations for evaluating measurement properties, this study examined the Catalan and Spanish versions of the ADAMS in adults with mild‐to‐severe ID and assessed internal consistency, test–retest reliability, measurement error, agreement, and internal structure through confirmatory factor analysis (CFA).

## 2 Methods

### 2.1 Study Population

Sample size was determined in accordance with COnsensus‐based Standards for the selection of health Measurement INstruments (COSMIN) guidelines for psychometric validation studies and recent recommendations suggesting approximately 5–10 participants per item and total samples of 200–300 participants (Mokkink et al. 2024; Prinsen et al. 2018). A total of 240 adults with mild‐to‐severe ID were recruited from six collaborating centres: two occupational therapy services, two occupational integration services, one residential service, and one sheltered work centre, using procedures comparable to those adopted in previous validation studies in adults with ID (Pett et al. 2021). Consecutive sampling from an initial pool of 280 eligible adults yielded 240 participants after application of the exclusion criteria, corresponding to a recruitment rate of 86%. Exclusion criteria were absence of signed informed consent from the participant or legal guardian, acute medical instability, and psychotropic medication changes within the previous 3 months. The study was approved by the Ethics and Research Committee of the Blanquerna School of Psychology, Education and Sport Sciences (Universitat Ramon Llull) and was conducted in accordance with the Declaration of Helsinki (identification code: 20210010D).

### 2.2 Instrument

#### 2.2.1 Anxiety, Depression and Mood Scale (ADAMS)

The ADAMS is a proxy‐rated instrument designed to assess emotional and behavioural problems over the previous 6 months in adults with ID. It comprises 28 items rated on a 4‐point Likert scale (0 = not occurred/not a problem; 3 = occurs a lot/severe problem) and five subscales: manic/hyperactive behaviour, depressed mood, social avoidance, general anxiety, and obsessive‐compulsive behaviour. Total scores range from 0 to 84, with higher scores indicating greater symptom severity. In the original validation study, the ADAMS demonstrated good internal consistency and test–retest reliability (Esbensen et al. 2003). The instrument was translated from English into Catalan and Spanish and culturally adapted for the present study using forward and back translation, expert review, and pilot testing with adults with ID (Beaton et al. 2000; McKown et al. 2020). Item content was then reviewed by linguists with expertise in Catalan, Spanish, and English, together with clinicians experienced in ID, to ensure semantic, idiomatic, and conceptual equivalence as well as clinical relevance (Teig et al. 2023). The Catalan and Spanish versions of the ADAMS are provided as online supplementary materials (Supplementary Materials 1 and 2).

### 2.3 Procedure

Representatives of the collaborating services, potential participants, and their legal guardians were invited to an information session where the aims of the study, study procedures, potential benefits, risks, and timelines were explained, following recommended approaches to informed consent in people with ID (Marín 2025).

Those who agreed to participate received written study information and an informed consent form and were given 5–10 days to consider participation before signing. For each participating adult with ID, the primary caregiver or legal guardian acted as the proxy informant. Proxy informants completed both the Catalan and Spanish versions of the ADAMS at baseline (T1) and again 7 days later (T2). At each time point, both versions were administered, with the order of administration randomised for each participant (Shimada and Katahira 2022). Test–retest reliability was assessed by comparing each language version with itself across T1 and T2. This interval was selected because it is short enough to minimise real symptom change while reducing recall bias (Mingins et al. 2024).

### 2.4 Statistical Analysis

Psychometric analyses focused on internal consistency, structural validity, test–retest reliability, measurement error, and agreement, in line with contemporary recommendations for evaluating measurement properties in health‐related instruments (Mokkink et al. 2024; Prinsen et al. 2018). Internal consistency for the ADAMS total score and subscales was assessed using Cronbach's alpha. Values of α≥0.70 were considered adequate for screening purposes, whereas values above 0.90 were interpreted cautiously because they may indicate item redundancy. Structural validity was examined using CFA. Because the ADAMS items are ordinal, CFA was conducted using polychoric correlations and robust weighted least squares mean and variance adjusted (WLSMV) estimation. Model fit was evaluated using the comparative fit index (CFI), Tucker–Lewis index (TLI), and root mean square error of approximation (RMSEA), with CFI/TLI ≥ 0.90 and RMSEA ≤ 0.08 considered acceptable, and CFI/TLI ≥ 0.95 and RMSEA ≤ 0.06 considered good fit. Given the heterogeneity of emotional and behavioural manifestations in adults with ID and the proxy‐rated nature of the ADAMS, perfect fit was not expected; CFA was therefore used to assess approximate structural validity. Test–retest reliability for ADAMS total and subscale scores was estimated using the concordance correlation coefficient (CCC) with 95% confidence intervals (Lin 1989, 1992; Mokkink et al. 2024). CCC values were interpreted as poor (< 0.50), moderate (0.50–0.75), good (0.75–0.90), and excellent (> 0.90). Absolute reliability was quantified using the standard error of measurement (SEM) and the smallest real difference (SRD). Relative errors were expressed as %SEM and %SRD relative to the maximum possible score for each scale. Agreement between T1 and T2 scores was further examined using Bland–Altman plots and the mean difference with 95% limits of agreement (Bland and Altman 1999; Prinsen et al. 2018).

Statistical analyses were conducted in R (R Core Team 2025), using lavaan for confirmatory factor analysis (Rosseel 2012), psych for descriptive and psychometric analyses, semTools for fit indices, irr for reliability analyses, and BlandAltmanLeh for agreement plots. Descriptive statistics were reported as means and standard deviations for normally distributed variables and medians with interquartile ranges for skewed variables. Missing data were below 5% and were handled using listwise deletion.

## 3 Results

### 3.1 Participant Characteristics

The sample comprised 240 adults with ID. Most proxy informants were legal guardians (56.3%), followed by parents (28.3%), work supervisors (12.9%), and direct support staff (2.5%). The mean length of the respondent‐participant relationship was 10.6 years (SD = 8.5), indicating long‐standing contact. Respondents most often had contact with participants in residential homes (37.9%) and day programmes (27.5%), with additional contact in community settings (9.2%), work environments (12.9%), and other contexts (12.5%).

Participants were predominantly female (57.9%) and had a mean age of 36.5 years (SD = 11.9). Most participants had mild ID (72.1%), followed by moderate (22.1%) and severe (3.8%) ID; severity was unknown for 2.1% of the sample. Most lived with their parents (81.7%), whereas smaller proportions lived in their own home without assistance (11.7%), in their own home with assistance (2.5%), or in supervised housing (4.2%). All participants had ID: 197 (82.1%) had ID only, 23 (9.6%) had co‐occurring autism spectrum disorder, and 20 (8.3%) had other co‐occurring disabilities. Psychometric analyses for the Catalan version were based on 236 participants because four T2 assessments were missing, whereas all 240 participants were included in the Spanish analyses.

### 3.2 Internal Consistency

Internal consistency of the ADAMS total score was acceptable in both versions (Catalan α=0.72; Spanish α=0.73), indicating similar reliability across languages. Subscale estimates are reported in Table 3.

### 3.3 Internal Structure

Confirmatory factor analysis of the original five‐factor ADAMS model, using polychoric correlations and WLSMV (weighted least squares mean and variance adjusted) estimation, showed acceptable absolute fit in both versions. RMSEA was 0.047 (95% CI: 0.039–0.055) for the Catalan version and 0.048 (95% CI: 0.039–0.056) for the Spanish version. Incremental fit was more modest, with CFI values of 0.821 and 0.832 and TLI values of 0.798 and 0.821 for the Catalan and Spanish versions, respectively (Table 1). These CFI and TLI values were below the predefined threshold of 0.90 for acceptable fit, indicating that evidence for structural validity based on incremental fit indices was limited. Inter‐factor correlations were high (r=0.65–0.85).

**TABLE 1:** Confirmatory factor analysis (CFA) fit indices: Catalan versus Spanish.

Fit index	Catalan	Spanish
*χ* ^2^ (df)	515 (339)	520 (339)
Baseline *χ* ^2^ (df)	1866 (378)	2069 (378)
RMSEA (95% CI)	0.047 (0.039–0.055)	0.048 (0.039–0.056)
RMSEA *p*	0.719	0.673
CFI	0.821	0.832
TLI	0.798	0.821

*Note: χ* ^2^ *p* < 0.001. WLSMV/polychoric. 95% CI for RMSEA. Full outputs: Supplementary Materials 3 (Catalan versions)‐Supplementary Materials 4 (Spanish versions).

The standardised factor loadings were broadly similar across the Catalan and Spanish versions, supporting comparable internal structure across languages (Table 2). Overall, the pattern of loadings was consistent with the proposed five‐factor model, although several items showed weak loadings and a small number of negative coefficients, especially in the social avoidance and Depression domains. All ADAMS items are scored in the same direction (0 = not a problem, 3 = severe problem); no items are reverse scored. The presence of both positive and negative standardised factor loadings therefore reflects the orientation of the latent factors and the model specification in the confirmatory factor analysis, rather than differences in item coding. We verified the coding and direction of all items and confirmed that they are consistent with the intended scoring of the ADAMS. Most standardised factor loadings were broadly similar across the Catalan and Spanish versions, although ADAMS16 showed a notable cross‐language discrepancy in direction and magnitude (Tang et al. 2025).

**TABLE 2:** Standardised factor loadings for the Catalan and Spanish versions of the ADAMS.

Item	Factor	Catalan (*β*)	Spanish (*β*)
ADAMS1	General anxiety	0.677	0.669
ADAMS3	General anxiety	−0.544	−0.558
ADAMS7	General anxiety	−0.663	−0.690
ADAMS11	General anxiety	−0.609	−0.602
ADAMS15	General anxiety	0.364	0.358
ADAMS24	General anxiety	−0.435	−0.437
ADAMS26	General anxiety	−0.339	−0.337
ADAMS2	Social avoidance	0.321	0.471
ADAMS6	Social avoidance	0.021	0.112
ADAMS13	Social avoidance	0.908	0.752
ADAMS19	Social avoidance	−0.194	−0.209
ADAMS21	Social avoidance	0.524	0.498
ADAMS25	Social avoidance	0.041	0.086
ADAMS27	Social avoidance	−0.233	−0.371
ADAMS4	Maniac/hyperactive behaviour	0.721	0.806
ADAMS12	Maniac/hyperactive behaviour	0.707	0.751
ADAMS17	Maniac/hyperactive behaviour	0.196	0.200
ADAMS22	Maniac/hyperactive behaviour	0.248	0.217
ADAMS3	Maniac/hyperactive behaviour	0.567	0.558
ADAMS5	Depressed mood	0.154	0.168
ADAMS9	Depressed mood	0.388	0.418
ADAMS10	Depressed mood	−0.522	−0.494
ADAMS14	Depressed mood	−0.106	−0.127
ADAMS18	Depressed mood	−0.244	−0.254
ADAMS23	Depressed mood	−0.462	−0.456
ADAMS28	Depressed mood	0.150	0.156
ADAMS8	Obsessive‐compulsive	0.470	0.593
ADAMS16	Obsessive‐compulsive	−0.933	0.910
ADAMS20	Obsessive‐compulsive	−0.573	−0.573

*Note: β* represents the standardised factor loading.

### 3.4 Test–Retest Reliability

Test–retest reliability was high across all ADAMS subscales in both versions (Table 3). Concordance correlation coefficients ranged from 0.87 to 0.96 in the Catalan version and from 0.91 to 0.96 in the Spanish version, indicating good to excellent temporal stability. Absolute measurement error was low, with SEM values ranging from 0.37 to 0.67 and SRD values from 1.02 to 1.87 across subscales. Relative error remained below 8% for most subscales, although it was higher for the obsessive‐compulsive subscale in both versions because of its narrower score range.

**TABLE 3:** Test–retest reliability of the ADAMS scale in Catalan and Spanish.

Version	Subscales	Mean1	SD1	Mean2	SD2	CCC (95% CI)	SEM	SRD	%SEM	%SRD	Cronbach's *α*
Catalan	Maniac/hyperactive behaviour (0–15)	5.60	2.38	5.68	2.42	0.92 (0.90–0.94)	0.67	1.87	7.99	8.29	0.642
	Depressed mood (0–21)	4.88	2.29	4.84	2.35	0.94 (0.92–0.95)	0.57	1.59	7.85	8.17	0.785
	Social avoidance (0–21)	5.56	2.67	5.42	2.61	0.96 (0.95–0.97)	0.51	1.43	6.22	6.50	0.318
	General anxiety (0–21)	6.26	2.73	6.24	2.79	0.94 (0.93–0.96)	0.66	1.83	7.02	7.31	0.651
	Obsessive‐compulsive (0–9)	2.30	1.24	2.33	1.19	0.87 (0.83–0.89)	0.44	1.22	12.74	13.21	0.377
	Total scale Cronbach's *α*										0.715
Spanish	Maniac/hyperactive behaviour (0–15)	5.60	2.33	5.68	2.31	0.92 (0.90–0.94)	0.65	1.80	7.68	7.96	0.678
	Depressed mood (0–21)	4.85	2.40	4.83	2.33	0.95 (0.93–0.96)	0.55	1.52	7.56	7.87	0.776
	Social avoidance (0–21)	5.53	2.61	5.42	2.79	0.96 (0.95–0.97)	0.52	1.44	6.30	6.57	0,303
	General anxiety (0–21)	6.35	2.80	6.31	2.82	0.95 (0.93–0.96)	0.65	1.81	6.87	7.15	0.694
	Obsessive‐compulsive (0–9)	2.28	1.23	2.30	1.22	0.91 (0.88–0.93)	0.37	1.02	10.77	11.18	0,333
	Total scale Cronbach's *α*										0.726

Abbreviations: 95% CI = confidence interval; CCC = concordance correlation coefficient; Cronbach's *α* = Cronbach's alpha; SD = standard deviation; SEM = standard error of measurement; SRD = smallest real difference.

### 3.5 Agreement Between Catalan and Spanish Versions (Bilingual Proxy Reports)

Among the 236 participants, 37 had both a Catalan and a Spanish ADAMS completed at baseline by their proxy informants (parents, legal guardians, or other caregivers). For these cases, we examined concordance between the two language versions using CCC and Bland–Altman analyses. CCC values ranged from 0.849 to 0.943 across subscales, indicating good to excellent agreement between the Catalan and Spanish versions (Table 4). Mean biases were small and 95% limits of agreement included zero for all subscales, suggesting no systematic bias between languages.

**TABLE 4:** Agreement between the Catalan and Spanish versions of the ADAMS in bilingual proxy reports (*n* = 37 proxy informants completing both language versions at baseline).

Subscale	CCC (95% CI)	Mean bias (95% CI)	95% limits of agreement
Maniac/hyperactive behaviour	0.849 (0.810–0.881)	0.104 (−0.068 to 0.276)	−2542 a 2750
Depressed mood	0.940 (0.923–0.953)	0.117 (−0.025 to 0.258)	−2064 a 2297
Social avoidance	0.919 (0.897–0.937)	0.050 (−0.084 to 0.184)	−2009 a 2109
General anxiety	0.928 (0.909–0.944)	0.029 (−0.131 to 0.189)	−2437 a 2495
Obsessive‐compulsive	0.851 (0.813–0.882)	−0.150 (−0.234 to −0.066)	−1442 a 1142
Total ADAMS	0.943 (0.928–0.956)	0.150 (−0.174 to 0.474)	−4839 a 5139

*Note:* CCC = concordance correlation coefficient. Mean bias = mean difference (Catalan−Spanish). Limits of agreement are the 95% limits from Bland–Altman analyses. Analyses are based on 37 proxy informants (parents, legal guardians, or other caregivers) who completed both language versions at baseline for the same participants with ID.

### 3.6 Bland–Altman Results

Bland–Altman analyses showed no evidence of systematic bias between T1 and T2 in either language version, with mean differences close to zero and 95% confidence intervals including zero across all subscales. Limits of agreement ranged from approximately ±2.0 to ±2.6 points, and no proportional bias was observed across the score range. Full Bland–Altman plots are provided in the Supplementary Material 5.

## 4 Discussion

This study examined the psychometric properties of the Catalan and Spanish versions of the ADAMS in adults with ID, extending prior validation work and providing evidence relevant to Spanish‐speaking contexts. Its main contribution is that the instrument appears to retain its value as a proxy‐rated screening measure while also illustrating the inherent limits of indirect assessment of internal states in this population and supporting only partial evidence of structural validity (Webb et al. 2024). In particular, although absolute fit indices were acceptable, incremental fit indices (CFI, TLI) did not meet the predefined criteria for acceptable structural validity; therefore, evidence for the five‐factor structure should be considered partial.

Our findings extend the existing evidence by showing that the ADAMS performs adequately in Catalan and Spanish adults with ID, with particularly strong temporal stability and acceptable internal consistency. This supports its use as a proxy‐based screening and monitoring tool in multilingual service settings despite only moderate support for the five‐factor structure (Esbensen et al. 2003; Hamers et al. 2019). Consistent with the CFA results, CFI and TLI values were below the 0.90 threshold predefined as indicative of acceptable fit, underscoring that structural validity evidence is limited. At the same time, the results suggest that the scale should be understood primarily as a practical clinical instrument rather than as a measure expected to reproduce perfectly separated latent domains in every sample or show complete cross‐language equivalence at the item level.

The relevance of this study is heightened by the shortage of validated emotional assessment tools for Spanish‐speaking adults with ID. A recent systematic review by González‐Sanguino et al. (2025) highlighted the limited availability of Spanish‐language instruments and the need for stronger psychometric evidence in adult ID populations, making this adaptation a meaningful contribution to the field and to clinical assessment in bilingual services (González‐Sanguino et al. 2025; Kumar et al. 2025; Patel et al. 2023). In this respect, the study addresses both a linguistic gap and a measurement gap with direct implications for routine practice.

A central interpretive issue is that emotional and behavioural difficulties in adults with ID rarely fall into clearly separated domains. Symptoms such as anxiety, low mood, social withdrawal, compulsive behaviour, and broader behavioural dysregulation often overlap, particularly when they are inferred from observation rather than directly reported by the person. The high inter‐factor correlations observed in the CFA are therefore not unexpected and should not be interpreted automatically as a weakness of the adapted scale, but rather as evidence that these domains are closely related in practice (Kildahl and Helverschou 2024). In other words, some degree of factor overlap is consistent with the clinical reality of the construct being measured. Within this broader pattern, ADAMS16 stood out because it showed opposite loadings across languages, which may indicate item‐level instability in the obsessive‐compulsive domain even when the overall factor structure was broadly comparable.

This matters because proxy and self‐report do not always converge, even when both are informative. In the ID literature, proxy ratings often differ from self‐ratings, and these differences should be treated as an expected feature of measurement rather than as simple error. Studies of quality‐of‐life ratings in adults with ID show that proxies frequently provide lower estimates than the individuals themselves, reinforcing the need for a nuanced interpretation of proxy data rather than dismissing it outright (Koch et al. 2015; Santoro et al. 2022; Webb et al. 2024). For that reason, the ADAMS should be read as a clinically useful indicator of emotional‐behavioural distress, not as a direct substitute for self‐report of internal experience or as evidence that all items function identically across language versions.

This interpretation is consistent with clinical guidance on psychiatric assessment in adults with ID, which emphasises the importance of multiple sources of information and careful contextual interpretation of behaviour. From that standpoint, the ADAMS is best understood as a screening tool that supports clinical decision‐making rather than as an instrument expected to yield perfectly distinct latent dimensions in every sample (Deb et al. 2022). That framing is more realistic and more useful for routine practice, especially in settings where caregivers or smupport staff are the main informants and where item‐level discrepancies may still occur.

The study also adds practical value by improving the interpretability of repeated measurement. When a tool is intended for follow‐up, internal consistency alone is not enough; clinicians also need to know whether observed change is likely to reflect genuine change rather than measurement noise. For that reason, agreement and measurement error are particularly relevant to real‐world use (Bland and Altman 1999; Geerinck et al. 2019; Hamers et al. 2019). The present findings therefore support the ADAMS not only as a screening instrument, but also as a tool that may help monitor symptoms over time in everyday services when interpreted cautiously.

The availability of both Catalan and Spanish versions is another important strength. Cross‐cultural adaptation is not simply a matter of translation, but of preserving conceptual equivalence and ensuring that the instrument remains meaningful and usable across contexts. In that respect, the study supports ADAMS as a potentially valuable resource in bilingual and multilingual service settings (Beaton et al. 2000; McKown et al. 2020). This is especially important in applied services, where consistency across versions can support both clinical care and research comparability despite the need for item‐level caution in ADAMS16.

More broadly, these findings sit within a wider clinical reality: emotional difficulties are common in people with ID, yet the field still lacks enough instruments with strong evidence for validity, interpretability, and practical applicability in routine care (Hughes‐McCormack et al. 2017; Leal and Hudson 2025). The ADAMS therefore contributes to a developing assessment landscape rather than to a fully resolved one. While reliability and temporal stability were good, structural validity evidence was limited by suboptimal CFI and TLI values, indicating that the five‐factor structure should be interpreted cautiously. Its value lies not in solving every measurement problem, but in offering a practical, adapted option where few robust tools are available for routine proxy‐based screening and monitoring.

Although the present study focuses on reliability and structural validity, these are fundamental first steps in the construct validation process. Future studies should therefore extend this work by examining criterion validity, inter‐rater reliability, test–retest reliability, and measurement invariance (Cook and Beckman 2006; Kildahl et al. 2025; Vetter and Cubbin 2019). Such work would also help determine whether alternative latent models better capture the organisation of emotional distress in ID, particularly given the item‐level discrepancy observed for ADAMS16.

### 4.1 Limitations

Several limitations should be noted. First, the ADAMS relies on proxy informants by design, which is appropriate in ID research and allows assessment when self‐report is not feasible; however, proxy ratings may still introduce interpretive uncertainty when the construct of interest is an internal emotional state (Santoro et al. 2022; Webb et al. 2024). Second, the study did not include concurrent criterion validation against psychiatric interview or another external standard, so the results should be interpreted as evidence of psychometric utility rather than diagnostic accuracy (Hermans and Evenhuis 2010). In addition, inter‐rater reliability was not assessed, which limits conclusions about the consistency of ratings across different informants (Ten Hove et al. 2024). Third, measurement invariance across language versions and subgroups was not tested, which limits conclusions about comparability across populations (Kildahl et al. 2025). Fourth, although all participants had ID, the sample was weighted toward mild ID and included relatively few individuals with severe ID, which limits the generalizability of the findings across the full spectrum of disability severity (Maulik et al. 2011; Schalock et al. 2021).

## 5 Conclusions

In summary, the Catalan and Spanish versions of the ADAMS appear to be useful proxy‐based tools for emotional screening and follow‐up in adults with ID. The study supports their clinical relevance and practical feasibility, while also indicating that evidence for the five‐factor structure is partial, as incremental fit indices (CFI, TLI) did not meet the predefined threshold of 0.90 for acceptable fit; their structure should therefore be interpreted cautiously and in the context of the broader complexities of mental health assessment in this population (Patel et al. 2023). The main strength of the instrument is not that it eliminates the challenges of proxy measurement, but that it provides a structured and clinically meaningful way to assess emotional distress when self‐report is limited. Overall, the Catalan and Spanish ADAMS versions are useful for proxy‐based screening and short‐term monitoring in adults with ID, but they should be interpreted as preliminary indicators that guide further clinical assessment rather than as diagnostic outcomes.

## Author Contributions

**Albert Oliveras‐Fàbregas:** conceptualization, data curation, formal analysis, funding acquisition, investigation, methodology, project administration, resources, software, validation, visualization, writing – original draft, writing – review and editing. **Salvador Simó‐Algado:** conceptualization, formal analysis, funding acquisition, methodology, supervision, validation, visualization, writing – original draft, writing – review and editing. **Raimon Milà‐Villaroel:** conceptualization, formal analysis, methodology, supervision, writing – original draft, writing – review and editing. **Josep Oliveras‐Cuquet:** conceptualization, formal analysis, methodology, writing – original draft, writing – review and editing. **Iván Sánchez‐Martínez:** conceptualization, formal analysis, methodology, writing – original draft, writing – review and editing. **Maria Giné‐Garriga:** conceptualization, data curation, formal analysis, funding acquisition, investigation, methodology, resources, software, supervision, validation, visualization, writing – original draft, writing – review and editing.

## Funding

This work was supported by the Col·legi de Fisioterapeutes de Catalunya (R06/21) and the Societat Catalano‐Balear de Fisioteràpia (2025 grant).

## Ethics Statement

The study was approved by the Ethics and Research Committee of the Blanquerna Faculty of Psychology, Educational Sciences and Sport (Universitat Ramon Llull) and was conducted in accordance with the Declaration of Helsinki (identification code: 20210010D).

## Consent

Informed consent was obtained from all participants involved in the study. Where applicable, consent was also obtained from legal guardians or representatives. All data are handled in accordance with the ethical approvals and the consent provided by the participants.

## Conflicts of Interest

The authors declare no conflicts of interest.

## Supporting information


**Supplementary Material 1** Resultats: structural equation models (Catalan versions).

**Supplementary Material 2** Resultats: structural equation models (Spanish versions).

**Supplementary Material 3** ESCALA PER L' ESTAT D'ÀNIM, LA DEPRESSIÓ I L'ANSIETAT (ADAMS) (Catalan version ADAMS).

**Supplementary Material 4** ESCALA PARA EL ESTADO DE ÁNIMO, LA DEPRESIÓN Y LA ANSIEDAD (ADAMS) (Spanish version ADAMS).

**Supplementary Material 5** Bland–Altman plots for test–retest agreement (T1 and T2) of the ADAMS scale in the Catalan and Spanish validations.

## Data Availability

The data that support the findings of this study are available on request from the corresponding author. The data are not publicly available due to privacy or ethical restrictions.
