# Supplementary material for: Psychometric Properties of the Catalan and Spanish Versions of the Anxiety, Depression and Mood Scale (ADAMS) in Adults With Intellectual Disabilities
Source: J Appl Res Intellect Disabil. 2026 Oct 22;39(6):e70323. doi: 10.1111/jar.70323 (PMC13691391; doi:10.1111/jar.70323)
Supplement: Supplementary file 1 [file JAR-39-e70323-s001.docx]

Bland–Altman plots for test–retest agreement (T1 and T2) of the ADAMS scale in the Catalan and Spanish validations

(a) Differences between the test and the repeat versus the mean of the two measurements of the average manic/hyperactive behavior in the Catalan version; (b) Differences between the test and the repeat versus the mean of the two measurements of the average depressed mood in the Catalan version; (c) Differences between the test and the repeat versus the mean of the two measurements of the average social avoidance in the Catalan version; (d) Differences between the test and the repeat versus the mean of the two measurements of the average general anxiety in the Catalan version; (e) Differences between the test and the repeat versus the mean of the two measurements of the average obsessive/compulsive behavior in the Catalan version; (f) Differences between the test and the repeat versus the mean of the two measurements of the average manic/hyperactive behavior in the Spanish version; (g) Differences between the test and the repeat versus the mean of the two measurements of the average depressed mood in the Spanish version; (h) Differences between the test and the repeat versus the mean of the two measurements of the average social avoidance in the Spanish version; (i) Differences between the test and the repeat versus the mean of the two measurements of the average general anxiety in the Spanish version; (j) Differences between the test and the repeat versus the mean of the two measurements of the average obsessive/compulsive behavior in the Spanish version.
