# Supplementary material for: Psychometric Properties of the Catalan and Spanish Versions of the Anxiety, Depression and Mood Scale (ADAMS) in Adults With Intellectual Disabilities
Source: J Appl Res Intellect Disabil. 2026 Oct 22;39(6):e70323. doi: 10.1111/jar.70323 (PMC13691391; doi:10.1111/jar.70323)
Supplement: Supplementary file 2 [file JAR-39-e70323-s002.docx]

ESCALA PARA EL ESTADO DE ÁNIMO, LA DEPRESIÓN Y LA ANSIEDAD
(ADAMS)

Participante :

Fecha:

**Información general de la persona objeto de evaluación**

**Género:**  Masculino  Femenino	**Edad:**

**Nivel de discapacidad intelectual:**

 Límite	 Leve	 Moderada	 Profunda	 Severa	 No lo sé

| **Situación residencial:** | Casa propia sin asistencia  Casa propia con asistencia  Comparte casa con sus padres  Piso tutelado  Reside con personal interno/en acogida  Otro tipo de residencia_______________ | Centro para grupos pequeños (<7 habitantes)  Centro para grupos medianos  (7-15 habitantes)  Asilo de ancianos  Instalación grande (colegio público/centro para discapacitados intelectuales/hospital >15 residentes) |
| --- | --- | --- |

**Discapacidades:**

 Deterioro del habla/lenguaje	 Trastornos psiquiátricos	 Discapacidad física

 Sordera/deficiencia auditiva	 Enfermedad crónica	 Autismo

 Discapacidad cerebral/neurológica	 Ceguera legal	 Discapacidad de aprendizaje

 Otros____________________

**Datos de la persona que rellena el formulario**

| **Su relación con esta persona:** | Personal de asistencia directa  Tutor/a  Padre/madre | Profesor/a Supervisor/a laboral  Pariente  Otro |
| --- | --- | --- |

**Duración de la relación:**	__________ años	__________ meses

| **Entorno de contacto:** | Comunidad  Programa de día | Hogar  Colegio | Trabajo  Otro _________ |
| --- | --- | --- | --- |

**Instrucciones**

La Escala para el Estado de Ánimo, la Depresión y la Ansiedad (ADAMS) incluye una lista de comportamientos que se pueden presentar en personas con discapacidad intelectual. Describa el comportamiento de esta persona durante los últimos 6 meses.

0	no se ha producido el comportamiento o este no supone un problema

1	comportamiento que se produce esporádicamente o que supone un problema menor

2	comportamiento que se produce con bastante frecuencia o que supone un problema moderado

3	comportamiento que se produce muchas veces o que supone un problema grave

|  | no supone  un problema | problema menor | problema moderado | problema grave |
| --- | --- | --- | --- | --- |
| 1. Nervioso/a | 0 | 1 | 2 | 3 |
| 2. Problemas para iniciar la comunicación | 0 | 1 | 2 | 3 |
| 3. No se relaja o calma | 0 | 1 | 2 | 3 |
| 4. Tiene periodos de excesode actividad | 0 | 1 | 2 | 3 |
| 5. Duerme más de lo normal | 0 | 1 | 2 | 3 |
| 6. Se aparta de los demás | 0 | 1 | 2 | 3 |
| 7. Tenso/a | 0 | 1 | 2 | 3 |
| 8. Presenta comportamientos ritualistas | 0 | 1 | 2 | 3 |
| 9. Estado de ánimo depresivo | 0 | 1 | 2 | 3 |
| 10. Triste | 0 | 1 | 2 | 3 |
| 11. Preocupado/a | 0 | 1 | 2 | 3 |
| 12. Ha mostrado dificultad a la hora de centrarse en una tarea o finalizar su trabajo | 0 | 1 | 2 | 3 |
| 13. Tímido/a | 0 | 1 | 2 | 3 |
| 14. Se fatiga con facilidad (por motivos distintos al sobrepeso) | 0 | 1 | 2 | 3 |
| 15. Ansioso/a | 0 | 1 | 2 | 3 |
| 16. Comprueba las cosas varias veces | 0 | 1 | 2 | 3 |
| 17. Se distrae con facilidad | 0 | 1 | 2 | 3 |
| 18. Le falta energía | 0 | 1 | 2 | 3 |
| 19. Evita a los demás, pasa la mayoría del tiempo solo/a | 0 | 1 | 2 | 3 |
| 20. Se enfada con facilidad si se interrumpen sus comportamientos ritualistas | 0 | 1 | 2 | 3 |
| 21. Su rostro no muestra emociones | 0 | 1 | 2 | 3 |
| 22. Ha mostrado dificultades a la hora de iniciar las tareas rutinarias | 0 | 1 | 2 | 3 |
| 23. Apático/a | 0 | 1 | 2 | 3 |
| 24. Sufre de ataques de pánico | 0 | 1 | 2 | 3 |
|  | no supone  un problema | problema menor | problema moderado | problema grave |
| 25. Evita el contacto visual | 0 | 1 | 2 | 3 |
| 26. Tiembla sin encontrarse en situaciones que le produzcan miedo | 0 | 1 | 2 | 3 |
| 27. Evita a sus iguales | 0 | 1 | 2 | 3 |
| 28. Tiende a llorar | 0 | 1 | 2 | 3 |

**Puntuación:**

| **Comportamiento maníaco/hiperactivo** | **Estado de ánimo depresivo** | **Evitación social** | **Ansiedad generalizada** | **Comportamiento obsesivo/compulsivo** |
| --- | --- | --- | --- | --- |
| 3. | 5. | 2. | 1. | 8. |
| 4. | 9. | 6. | 3. | 16. |
| 12. | 10. | 13. | 7. | 20. |
| 17. | 14. | 19. | 11. |  |
| 22. | 18. | 21. | 15. |  |
|  | 23. | 25. | 24. |  |
|  | 28. | 27. | 26. |  |
| **Total:** | **Total:** | **Total:** | **Total:** | **Total:** |
