# Supplementary material for: Psychometric Properties of the Catalan and Spanish Versions of the Anxiety, Depression and Mood Scale (ADAMS) in Adults With Intellectual Disabilities
Source: J Appl Res Intellect Disabil. 2026 Oct 22;39(6):e70323. doi: 10.1111/jar.70323 (PMC13691391; doi:10.1111/jar.70323)
Supplement: Supplementary file 3 [file JAR-39-e70323-s003.docx]

ESCALA PER L’ ESTAT D’ÀNIM, LA DEPRESSIÓ I L’ANSIETAT(ADAMS)

Participant :

Dia:

**Informació general de la persona objecte d’avaluació**

**Gènere:**	 Masculí	 Femení	**Edat:**

**Nivell de discapacitat intel·lectual:**  Límit	 Moderada

 Profunda	 Lleu

 Severa	 No ho sé

| **Situació residencial:** | Casa pròpia sense assistència  Casa pròpia amb assistència  Comparteix casa amb els pares  Pis tutelat  Resideix amb personal intern/en acolliment  Altres tipus de residència_______________ | Centre per grups petits (<7 habitants)  Centre per grups mitjans (7-15 habitants)  Asil d’avis  Instalació gran (col.legi públic/centre per discapacitats intelectuals/hospital >15 residents) |
| --- | --- | --- |

**Discapacitats:**  Deteriore de la parla/llenguatg	 Trastorns psiquiàtrics

 Discapacitat física	 Sordesa/deficiència auditiva

 Malaltia crónica	 Autisme

 Discapacitat cerebral/neurològica	 Ceguera legal

Discapacitat d´aprenantatge	 Altres

**Dades de la persona que omple el formulari**

| **La seva relació amb aquesta persona:** | Personal d’assistència directe  Tutor/a  Pare/mare | Professor/a Supervisor/a laboral  Parent  Altres |
| --- | --- | --- |

**Duració de la relació:**	__________ anys	__________ mesos

| **Entorn del contacte:** | Comunitat  Programa de dia | Llar  Col·legi | Treball  Altres _________ |
| --- | --- | --- | --- |

**Instruccions**

L’Escala per l’Estat d’Ànim, la Depresió i l’Ansietat (ADAMS) inclou una llista de comportaments que es poden presentar en persones amb discapacitat intel·lectual. Descriu el comportament d’aquesta persona durant els últims 6 mesos.

0	no es produeix el comportament o aquest no suposa un problema

1	comportament que es produeix esporádicament o que suposa un problema menor

2	comportament que es produeix bastant frecuent o que supone un problema moderat

3	comportament que es produeix moltes vegades o que suposa un problema greu

|  | no suposa un problema | problema menor | problema moderat | problema greu |
| --- | --- | --- | --- | --- |
| 1. Nerviós/a | 0 | 1 | 2 | 3 |
| 2. Problemes per iniciar la comunicació | 0 | 1 | 2 | 3 |
| 3. No es relaxa o calma | 0 | 1 | 2 | 3 |
| 4. Presenta períodes d’excés d’activitat | 0 | 1 | 2 | 3 |
| 5. Dorm més del normal | 0 | 1 | 2 | 3 |
| 6. S’allunya dels demés | 0 | 1 | 2 | 3 |
| 7. Tens/a | 0 | 1 | 2 | 3 |
| 8. Presenta comportaments ritualistes | 0 | 1 | 2 | 3 |
| 9. Estat d’ànim depressiu | 0 | 1 | 2 | 3 |
| 10. Trist | 0 | 1 | 2 | 3 |
| 11. Preocupat/da | 0 | 1 | 2 | 3 |
| 12. Ha mostrat dificultat a l’hora de centrar-se en una tasca o finalitzar la seva feina | 0 | 1 | 2 | 3 |
| 13. Tímid/da | 0 | 1 | 2 | 3 |
| 14. Es fatiga amb facilitat (per motius diferents al sobrepès) | 0 | 1 | 2 | 3 |
| 15. Ansiós/a | 0 | 1 | 2 | 3 |
| 16. Comproba les coses diverses vegades | 0 | 1 | 2 | 3 |
| 17. Es distreu amb facilitat | 0 | 1 | 2 | 3 |
| 18. Li falta energia | 0 | 1 | 2 | 3 |
| 19. Evita els demés, passa la majoria del temps sol/a | 0 | 1 | 2 | 3 |
| 20. S’enfada amb facilitat si s’interrumpeix els seus comportaments ritualistes | 0 | 1 | 2 | 3 |
| 21. El seu rostre no mostra emocions | 0 | 1 | 2 | 3 |
| 22. Ha mostrat dificultats a l’hora d’iniciar les tasques habituals | 0 | 1 | 2 | 3 |
|  | no suposa un problema | problema menor | problema moderat | problema greu |
| 23. Apàtic/a | 0 | 1 | 2 | 3 |
| 24. Pateix atacs de pànic | 0 | 1 | 2 | 3 |
|  |  |  |  |  |
| 25. Evita el contacte visual | 0 | 1 | 2 | 3 |
| 26. Tremola sense trobar-se en situacions que li produeixen por | 0 | 1 | 2 | 3 |
| 27. Evita els seus iguals | 0 | 1 | 2 | 3 |
| 28. Tendeix a plorar | 0 | 1 | 2 | 3 |

**Puntuació:**

| **Comportament maniac/hiperactiu** | **Estad d’ànim depressiu** | **Evitació social** | **Ansietat generalitzada** | **Comportament obsessiu/compulsiu** |
| --- | --- | --- | --- | --- |
| 3. | 5. | 2. | 1. | 8. |
| 4. | 9. | 6. | 3. | 16. |
| 12. | 10. | 13. | 7. | 20. |
| 17. | 14. | 19. | 11. |  |
| 22. | 18. | 21. | 15. |  |
|  | 23. | 25. | 24. |  |
|  | 28. | 27. | 26. |  |
| **Total:** | **Total:** | **Total:** | **Total:** | **Total:** |
