# Supplementary material for: Psychometric Properties of the Catalan and Spanish Versions of the Anxiety, Depression and Mood Scale (ADAMS) in Adults With Intellectual Disabilities
Source: J Appl Res Intellect Disabil. 2026 Oct 22;39(6):e70323. doi: 10.1111/jar.70323 (PMC13691391; doi:10.1111/jar.70323)
Supplement: Supplementary file 4 [file JAR-39-e70323-s004.docx]

# Resultats

**Structural Equation Models**

Models Info

**Estimation Method	DWLS**

**Optimization Method	NLMINB**

**Number of observations**	**236**

**Free parameters**	**123**

**Standard errors	Robust**

**Scaled test	Mean adjusted scaled and shifted**

**Converged	TRUE**

**Iterations**	**23**

**Model	Anxiety =~ ADAMS1 + ADAMS3 + ADAMS7 + ADAMS11 + ADAMS15 + ADAMS24 + ADAMS26 Social =~ ADAMS2 + ADAMS6 + ADAMS13 + ADAMS19 + ADAMS21 + ADAMS25 + ADAMS27 Manic =~ ADAMS4 + ADAMS12 + ADAMS17 + ADAMS22 + ADAMS3**

**Depression =~ ADAMS5 + ADAMS9 + ADAMS10 + ADAMS14 + ADAMS18 + ADAMS23 + ADAMS28 OCD =~ ADAMS8 + ADAMS16 + ADAMS20**

Variable (ADAMS1,ADAMS3,ADAMS7,ADAMS11,ADAMS15,ADAMS24,ADAMS26,ADAMS2,ADAMS6,ADAMS13,ADAMS19,ADAMS21,ADAMS25,ADAMS27,ADAMS4,ADAMS12,ADAMS17,ADAMS22,ADAMS5

has been coerced to ordered type.

## Overall Tests

| Model tests |  | | |
| --- | --- | --- | --- |
| **Label** | **X²** | **df** | **p** |
| User Model | 515 | 339 | <.001 |
| Baseline Model | 1866 | 378 | <.001 |
| Scaled User | 478 | 339 | <.001 |
| Scaled Baseline | 1160 | 378 | <.001 |

Fit indices

**95% Confidence Intervals**

| **Type** | **SRMR** | **RMSEA** | **Lower** | **Upper** | **RMSEA p** |
| --- | --- | --- | --- | --- | --- |
| Classical | 0.084 | 0.047 | 0.039 | 0.055 | 0.719 |
| Robust | 0.077 | 0.064 | 0.054 | 0.074 | 0.014 |
| Scaled | 0.077 | 0.042 | 0.033 | 0.050 | 0.945 |

| User model versus baseline model |  | | |
| --- | --- | --- | --- |
|  | **Model** | **Scaled** | **Robust** |
| **Comparative Fit Index (CFI)** | 0.821 | 0.782 | 0.685 |
| **Tucker-Lewis Index (TLI)** | 0.798 | 0.752 | 0.648 |
| **Bentler-Bonett Non-normed Fit Index (NNFI)** | 0.808 | 0.802 | 0.696 |
| **Relative Noncentrality Index (RNI)** | 0.821 | 0.782 | 0.685 |
| **Bentler-Bonett Normed Fit Index (NFI)** | 0.654 | 0.528 |  |
| **Bollen's Relative Fit Index (RFI)** | 0.602 | 0.490 |  |
| **Bollen's Incremental Fit Index (IFI)** | 0.825 | 0.792 |  |
| **Parsimony Normed Fit Index (PNFI)** | 0.599 | 0.487 |  |

## Estimates

**95% Confidence Intervals**

| **Label** | **Latent** | **Observed** | **Estimate** | **SE** | **Lower** | **Upper** | **β** | **z** | **p** |
| --- | --- | --- | --- | --- | --- | --- | --- | --- | --- |
| p1 | Anxiety | ADAMS1 | 0.6773 | 0.0529 | 0.57358 | 0.7809 | 0.6773 | 12.803 | <.001 |
| p2 |  | ADAMS3 | -0.5439 | 0.0629 | -0.66715 | -0.4206 | -0.5439 | -8.646 | <.001 |
| p3 |  | ADAMS7 | -0.6631 | 0.0483 | -0.75776 | -0.5685 | -0.6631 | -13.735 | <.001 |
| p4 |  | ADAMS11 | -0.6094 | 0.0563 | -0.71975 | -0.4989 | -0.6094 | -10.818 | <.001 |
| p5 |  | ADAMS15 | 0.3636 | 0.0686 | 0.22908 | 0.4980 | 0.3636 | 5.299 | <.001 |
| p6 |  | ADAMS24 | -0.4345 | 0.0693 | -0.57035 | -0.2987 | -0.4345 | -6.271 | <.001 |
| p7 |  | ADAMS26 | -0.3390 | 0.0735 | -0.48313 | -0.1949 | -0.3390 | -4.610 | <.001 |
| p8 | Social | ADAMS2 | 0.3205 | 0.0817 | 0.16028 | 0.4807 | 0.3205 | 3.921 | <.001 |
| p9 |  | ADAMS6 | 0.0212 | 0.0924 | -0.15995 | 0.2023 | 0.0212 | 0.229 | 0.819 |
| p10 |  | ADAMS13 | 0.9080 | 0.1051 | 0.70210 | 1.1139 | 0.9080 | 8.643 | <.001 |
| p11 |  | ADAMS19 | -0.1940 | 0.0938 | -0.37786 | -0.0102 | -0.1940 | -2.069 | 0.039 |
| p12 |  | ADAMS21 | 0.5243 | 0.0798 | 0.36778 | 0.6807 | 0.5243 | 6.567 | <.001 |
| p13 |  | ADAMS25 | 0.0414 | 0.0977 | -0.14997 | 0.2329 | 0.0414 | 0.424 | 0.671 |
| p14 |  | ADAMS27 | -0.2329 | 0.0824 | -0.39446 | -0.0713 | -0.2329 | -2.825 | 0.005 |
| p15 | Manic | ADAMS4 | 0.7214 | 0.0565 | 0.61073 | 0.8321 | 0.7214 | 12.773 | <.001 |
| p16 |  | ADAMS12 | 0.7067 | 0.0579 | 0.59324 | 0.8201 | 0.7067 | 12.212 | <.001 |
| p17 |  | ADAMS17 | 0.1963 | 0.0760 | 0.04739 | 0.3453 | 0.1963 | 2.584 | 0.010 |
| p18 |  | ADAMS22 | 0.2475 | 0.0793 | 0.09211 | 0.4029 | 0.2475 | 3.122 | 0.002 |
| p19 |  | ADAMS3 | 0.5666 | 0.0630 | 0.44311 | 0.6901 | 0.5666 | 8.991 | <.001 |
| p20 | Depression | ADAMS5 | 0.1537 | 0.0827 | -0.00851 | 0.3159 | 0.1537 | 1.857 | 0.063 |
| p21 |  | ADAMS9 | 0.3878 | 0.0974 | 0.19685 | 0.5786 | 0.3878 | 3.981 | <.001 |
| p22 |  | ADAMS10 | -0.5218 | 0.1013 | -0.72044 | -0.3232 | -0.5218 | -5.150 | <.001 |
| p23 |  | ADAMS14 | -0.1061 | 0.1019 | -0.30573 | 0.0936 | -0.1061 | -1.041 | 0.298 |
| p24 |  | ADAMS18 | -0.2438 | 0.0931 | -0.42629 | -0.0612 | -0.2438 | -2.617 | 0.009 |
| p25 |  | ADAMS23 | -0.4620 | 0.0874 | -0.63337 | -0.2907 | -0.4620 | -5.286 | <.001 |
| p26 |  | ADAMS28 | 0.1501 | 0.0880 | -0.02237 | 0.3225 | 0.1501 | 1.706 | 0.088 |
| p27 | OCD | ADAMS8 | 0.4704 | 0.0741 | 0.32511 | 0.6157 | 0.4704 | 6.346 | <.001 |
| p28  p29 |  | ADAMS16  ADAMS20 | -0.9327  -0.5723 | 0.1198  0.0721 | -1.16759  -0.7143 | -0.6978  -0.4316 | -0.9327  -0.5729 | -7.783  -7.943 | <.001  <.001 |
|  |  |  |  |  |  |  |  |  |  |

## Path Model

### Path diagrams
