# Supplementary material for: Psychometric Properties of the Catalan and Spanish Versions of the Anxiety, Depression and Mood Scale (ADAMS) in Adults With Intellectual Disabilities
Source: J Appl Res Intellect Disabil. 2026 Oct 22;39(6):e70323. doi: 10.1111/jar.70323 (PMC13691391; doi:10.1111/jar.70323)
Supplement: Supplementary file 5 [file JAR-39-e70323-s005.docx]

# Resultats

**Structural Equation Models**

Models Info

**Estimation Method	DWLS**

**Optimization Method	NLMINB**

**Number of observations**	**236**

**Free parameters**	**123**

**Standard errors	Robust**

**Scaled test	Mean adjusted scaled and shifted**

**Converged	TRUE**

**Iterations**	**21**

**Model	Anxiety =~ ADAMS1 + ADAMS3 + ADAMS7 + ADAMS11 + ADAMS15 + ADAMS24 + ADAMS26 Social =~ ADAMS2 + ADAMS6 + ADAMS13 + ADAMS19 + ADAMS21 + ADAMS25 + ADAMS27 Manic =~ ADAMS4 + ADAMS12 + ADAMS17 + ADAMS22 + ADAMS3**

**Depression =~ ADAMS5 + ADAMS9 + ADAMS10 + ADAMS14 + ADAMS18 + ADAMS23 + ADAMS28 OCD =~ ADAMS8 + ADAMS16 + ADAMS20**

Variable (ADAMS1,ADAMS3,ADAMS7,ADAMS11,ADAMS15,ADAMS24,ADAMS26,ADAMS2,ADAMS6,ADAMS13,ADAMS19,ADAMS21,ADAMS25,ADAMS27,ADAMS4,ADAMS12,ADAMS17,ADAMS22,ADAMS5

has been coerced to ordered type.

## Overall Tests

| Model tests |  | | |
| --- | --- | --- | --- |
| **Label** | **X²** | **df** | **p** |
| User Model | 520 | 339 | <.001 |
| Baseline Model | 2069 | 378 | <.001 |
| Scaled User | 488 | 339 | <.001 |
| Scaled Baseline | 1250 | 378 | <.001 |

Fit indices

**95% Confidence Intervals**

| **Type** | **SRMR** | **RMSEA** | **Lower** | **Upper** | **RMSEA p** |
| --- | --- | --- | --- | --- | --- |
| Classical | 0.084 | 0.048 | 0.039 | 0.056 | 0.673 |
| Robust | 0.076 | 0.068 | 0.058 | 0.078 | 0.002 |
| Scaled | 0.076 | 0.043 | 0.034 | 0.052 | 0.908 |

| User model versus baseline model |  | | |
| --- | --- | --- | --- |
|  | **Model** | **Scaled** | **Robust** |
| **Comparative Fit Index (CFI)** | 0.832 | 0.809 | 0.623 |
| **Tucker-Lewis Index (TLI)** | 0.821 | 0.780 | 0.591 |
| **Bentler-Bonett Non-normed Fit Index (NNFI)** | 0.810 | 0.763 | 0.651 |
| **Relative Noncentrality Index (RNI)** | 0.813 | 0.789 | 0.703 |
| **Bentler-Bonett Normed Fit Index (NFI)** | 0.719 | 0.610 |  |
| **Bollen's Relative Fit Index (RFI)** | 0.670 | 0.565 |  |
| **Bollen's Incremental Fit Index (IFI)** | 0.835 | 0.801 |  |
| **Parsimony Normed Fit Index (PNFI)** | 0.611 | 0.547 |  |

## Estimates

**95% Confidence Intervals**

| **Label** | **Latent** | **Observed** | **Estimate** | **SE** | **Lower** | **Upper** | **β** | **z** | **p** |
| --- | --- | --- | --- | --- | --- | --- | --- | --- | --- |
| p1 | Anxiety | ADAMS1 | 0.6688 | 0.0542 | 0.5626 | 0.7751 | 0.6688 | 12.336 | <.001 |
| p2 |  | ADAMS3 | -0.5576 | 0.0587 | -0.6727 | -0.4426 | -0.5576 | -9.499 | <.001 |
| p3 |  | ADAMS7 | -0.6897 | 0.0474 | -0.7826 | -0.5969 | -0.6897 | -14.556 | <.001 |
| p4 |  | ADAMS11 | -0.6023 | 0.0579 | -0.7157 | -0.4889 | -0.6023 | -10.411 | <.001 |
| p5 |  | ADAMS15 | 0.3581 | 0.0704 | 0.2201 | 0.4961 | 0.3581 | 5.085 | <.001 |
| p6 |  | ADAMS24 | -0.4371 | 0.0680 | -0.5704 | -0.3037 | -0.4371 | -6.425 | <.001 |
| p7 |  | ADAMS26 | -0.3371 | 0.0740 | -0.4821 | -0.1920 | -0.3371 | -4.553 | <.001 |
| p8 | Social | ADAMS2 | 0.4711 | 0.0803 | 0.3137 | 0.6286 | 0.4711 | 5.865 | <.001 |
| p9 |  | ADAMS6 | 0.1120 | 0.1011 | -0.0862 | 0.3102 | 0.1120 | 1.107 | 0.268 |
| p10 |  | ADAMS13 | 0.7519 | 0.0728 | 0.6092 | 0.8946 | 0.7519 | 10.324 | <.001 |
| p11 |  | ADAMS19 | -0.2093 | 0.0957 | -0.3968 | -0.0217 | -0.2093 | -2.187 | 0.029 |
| p12 |  | ADAMS21 | 0.4982 | 0.0738 | 0.3535 | 0.6430 | 0.4982 | 6.748 | <.001 |
| p13 |  | ADAMS25 | 0.0857 | 0.0959 | -0.1023 | 0.2737 | 0.0857 | 0.893 | 0.372 |
| p14 |  | ADAMS27 | -0.3706 | 0.0784 | -0.5244 | -0.2169 | -0.3706 | -4.726 | <.001 |
| p15 | Manic | ADAMS4 | 0.8056 | 0.0554 | 0.6971 | 0.9141 | 0.8056 | 14.551 | <.001 |
| p16 |  | ADAMS12 | 0.7508 | 0.0550 | 0.6430 | 0.8587 | 0.7508 | 13.647 | <.001 |
| p17 |  | ADAMS17 | 0.2001 | 0.0718 | 0.0594 | 0.3407 | 0.2001 | 2.787 | 0.005 |
| p18 |  | ADAMS22 | 0.2173 | 0.0778 | 0.0649 | 0.3697 | 0.2173 | 2.794 | 0.005 |
| p19 |  | ADAMS3 | 0.5575 | 0.0628 | 0.4345 | 0.6805 | 0.5575 | 8.881 | <.001 |
| p20 | Depression | ADAMS5 | 0.1682 | 0.0855 | 7.08e-4 | 0.3357 | 0.1682 | 1.968 | 0.049 |
| p21 |  | ADAMS9 | 0.4176 | 0.0940 | 0.2334 | 0.6018 | 0.4176 | 4.443 | <.001 |
| p22 |  | ADAMS10 | -0.4935 | 0.1007 | -0.6908 | -0.2961 | -0.4935 | -4.901 | <.001 |
| p23 |  | ADAMS14 | -0.1269 | 0.1024 | -0.3276 | 0.0738 | -0.1269 | -1.239 | 0.215 |
| p24 |  | ADAMS18 | -0.2536 | 0.0927 | -0.4352 | -0.0719 | -0.2536 | -2.736 | 0.006 |
| p25 |  | ADAMS23 | -0.4555 | 0.0881 | -0.6281 | -0.2830 | -0.4555 | -5.174 | <.001 |
| p26 |  | ADAMS28 | 0.1557 | 0.0879 | -0.0166 | 0.3280 | 0.1557 | 1.771 | 0.077 |
| p27 | OCD | ADAMS8 | 0.5933 | 0.0757 | 0.4449 | 0.7416 | 0.5933 | 7.839 | <.001 |
| p28 |  | ADAMS16 | 0.9099 | 0.1022 | 0.7096 | 1.1103 | 0.9099 | 8.901 | <.001 |
| p29 |  | ADAMS20 | -0.5729 | 0.0721 | -0.7143 | -0.4316 | -0.5729 | -7.943 | <.001 |

## Path Model

### Path diagrams
